# Spectrum of Biliary Lesions/Neoplasms in Hepatic Parenchyma with Reference to a Precursor of Small Duct-Type Intrahepatic Cholangiocarcinoma: Comprehensive Categorization into Three Groups

**DOI:** 10.3390/cancers18020328

**Published:** 2026-01-21

**Authors:** Yasuni Nakanuma, Motoko Sasaki, Yuko Kakuda, Takuma Oishi

**Affiliations:** 1 Division of Pathology, Shizuoka Cancer Center, Shizuoka 411-8777, Japan; y.kakuda@scchr.jp (Y.K.); ta.oishi@scchr.jp (T.O.); 2 Department of Diagnostic Pathology, Fukui Prefecture Saiseikai Hospital, Wadanakacho Funahashi 7-1, Fukui 918-8503, Japan; 3 Department of Human Pathology, Graduate School of Medicine, Kanazawa University, Kanazawa 920-8640, Japan; m8sasaki@med.kanazawa-u.ac.jp

**Keywords:** precursors, intrahepatic cholangiocarcinoma, small duct-type, benign biliary lesions/neoplasms, von Meyenburg complex, ductal plate malformation, bile duct adenoma, biliary adenofibroma

## Abstract

Intrahepatic cholangiocarcinomas (iCCAs) are histologically subdivided into small duct-type (SD-iCCA) and large duct-type (LD-iCCA). LD-iCCA versus SD-iCCA may differ in the molecular/genetic profiles and oncogenesis, including precursor lesions. While several precursors, such as high-grade biliary intraepithelial neoplasm (BilIN) and intraductal papillary neoplasm of bile duct (IPNB), have been proposed for LD-iCCA, the potential SD-iCCA precursors remain to be identified. Amid growing interests in the precursors of SD-iCCA, benign “biliary lesions/neoplasms developing in the hepatic parenchyma (BLNP)” such as von Meyenburg complexes (VMCs), bile duct adenomas (BDAs), and biliary adenofibroma (BAF), have been noted to determine whether they have the potential for precursor of SD-iCCA. Herein, these BLNPs were reviewed. BLNP can be classified into three categories. First, traditional VMC and BDA in normal livers which lack atypical features are categorized as “traditional BLNP”. Second, a constellation of several lesions such as VMC and BDA detectable in the background livers of SD-iCCA and in chronic liver disease (unusual VMC and BDA), VMC with dysplastic features, BDA located in the deep hepatic parenchyma, multiple BDA, BDA presenting the *BRAF V600E* mutation, and BAF harboring variable dysplasia or in situ carcinomas, which may include neoplastic lesions but do not show invasive growth, are categorized as “unusual/dysplastic BLNP”. Third, tubulocystic carcinoma with BAF-like features (AI-TCC) and SD-iCCA with ductal plate malformation (DPMP) which share overlapping features and show relatively good post-operative outcomes and retained features of VMC or DPM, and BDA and BAF, are categorized as “low-grade malignant BLNP”. While the first category is benign and may not be related to SD-iCCA, some of the second category may be related to SD-iCCA, and the third category is malignant and shows invasive growth. The latter two categories may form a common biliary tumorigenic spectrum involving BLNP. Precursors of SD-iCCA, if they exist, may be included in the second category, and the third category may represent unique carcinomas possibly associated with or followed by conventional SD-iCCA. In conclusion, this novel approach to categorize BLNPs into three categories guarantees further studies of precursors of and their progression to conventional SD-iCCA.

## Simple Summary

Benign “biliary lesions/neoplasms developing in the hepatic parenchyma (BLNP)” were reviewed with reference to potential SD-iCCA precursors and can be classified into three categories. (i) Traditional VMC and BDA are categorized as “traditional BLNP”. (ii) A constellation of several lesions such as VMC and BDA detectable in the background livers of SD-iCCA, VMC with dysplastic features, and BAF harboring variable dysplasia are categorized as “unusual/dysplastic BLNP”. (iii) Tubulocystic carcinoma with BAF-like features (AI-TCC) and SD-iCCA with ductal plate malformation (DPMP) are categorized as “low-grade malignant BLNP”. While the first category is benign, some of the second category may be related to SD-iCCA, and the third category is malignant. Precursors of SD-iCCA may be included in the second category, and the third category may represent unique carcinomas possibly associated with conventional SD-iCCA. This novel approach guarantees further studies of precursors of and their progression to conventional SD-iCCA.

## 1. Introduction

Intrahepatic cholangiocarcinoma (iCCA) is a devastating malignant type of tumor, accounting for 5–15% of all primary liver tumors, and its incidence and mortality rates are now increasing worldwide [1,2,3,4,5]. It may arise de novo or from biliary epithelial lesions or neoplasms (multistep mechanisms) [6,7,8,9]. The heterogeneity of iCCA with regard to its clinical features, pathological findings, and molecular/genetic alterations has recently been revealed, reflecting the diverse phenotypes of cholangiocytes along the biliary tree from which iCCAs have arisen [10,11], and iCCAs can be subdivided into small duct-type iCCA (SD-iCCA) and large duct-type iCCA (LD-iCCA). LD-iCCA is located in the proximity of hepatic hilar regions and arises in the lumen and peribiliary glands of large bile ducts, characterized by infiltrative neoplastic glands lined by mucin-positive columnar cells in desmoplastic stroma with more aggressive behaviors [1,7,8,12,13,14]. They shared many histologic/molecular features of perihilar and distal (p/d) CCA [4,8,15,16], and they could also be regarded as the biliary counterparts of pancreatic ductal adenocarcinomas [17,18]. By contrast, SD-iCCA appears to be a cancerous counterpart of hepatic parenchymal biliary ductules or hepatocytes or hepatic progenitor cells, typically arising within and invading the hepatic parenchyma, and usually presenting grossly as a mass-forming (MF) tumor [1,4,9]. SD-iCCA is composed of small glands/tubules with scant to absent mucin production, and often has a pushing border with hypocellular sclerotic stroma [1,4,6,12,15]. SD-iCCA does not infrequently arise in chronic advanced liver diseases, while LD-iCCA is occasionally associated with chronic biliary tract diseases such as hepatolithiasis and primary sclerosing cholangitis [1,4,7,8,16]. Mutations of *p53*, *K-RAS*, and *SMAD4* were frequently reported in LD-iCCA; however, SD-iCCA had different mutation profiles, such as low *K-RAS* mutation and high *IDH1/IDH2*, *ARID1A* and *BAP1* mutations, and *FGFR* fusions, and amplification in genes, such as *EGR2*, *CCDN1,* and *CCNE1* [3,12,14,19,20,21].

Interestingly, LD-iCCA versus SD-iCCA may also differ in the oncogenesis, including precursor lesions [6,9,12,13,21,22,23]. Indeed, the former may derive from precursors such as high-grade biliary intraepithelial neoplasm (high-grade BilIN) and intraductal papillary neoplasm of the bile duct (IPNB) identifiable in the lumen and peribiliary glands of the large bile ducts through a stepwise pathogenesis, and the basic concept of these precursor lesions and carcinogenesis of LD-iCCA may be similar to the general concepts of “dysplasia-carcinoma sequence” or “adenoma-carcinoma sequence” being elucidated in carcinogenesis in the pancreas and gastrointestinal tract [7,8,18,24]. In contrast, the identification of potential precursors and oncogenesis of SD-iCCA remains only speculative and controversial [6,9,22,25]. Amid growing interests in precursor lesions of SD-iCCA, so-called benign “biliary lesions/neoplasms in the hepatic parenchyma,” which have been regarded as reactive changes or remnant developmental anomalies so far, have been noted to have potential for precursor lesions of SD-iCCA [6,9,22,23,25,26].

Several kinds of benign non-hepatocytic, biliary lesions/neoplasms have been reported in the hepatic parenchyma, and they commonly express phenotypes of bile ductules and small bile ducts but not those of large bile ducts [6,25,26,27,28,29] (Table 1). Some benign biliary solid and solid/cystic lesions, such as von Meyenburg complexes (VMCs) and bile duct adenomas (BDAs), are complicated by invasive SD-iCCA [25,26,30,31]. Biliary adenofibroma (BAF), which used to be classified as a benign biliary neoplasm [1,32], always presents mild to marked atypical lesions, and is frequently associated with invasive carcinoma, and could be regarded as an SD-iCCA precursor [33,34,35,36]. Instead, other benign, biliary cystic lesions in the hepatic parenchyma, such as simple hepatic cysts, polycystic livers, and multicystic biliary hamartoma (MCBH), are not or incidentally complicated with CCA [10,28,29]. Non-hepatocellular and non-biliary benign diseases—such as endometric cysts and hepatic foregut cysts, which are regarded as ectopic or heterotopic lesions—are not associated with CCA, but these diseases can be complicated with squamous or endometrioid carcinoma [10,27]. The latter two groups will not be discussed here.

The biologic and clinical behaviors of VMC, BDA, and BAF, and their relations to biliary malignancy have been reported in the literature [6,9,26,27,34] (Table 2), though the histologic definition and diagnostic criteria of these lesions does not seem the same in the literature, and the significance and spectrum of these three lesions referring to the malignant transformation remains controversial and unsettled [6,9,25,26,27,37].

Herein, we will review the updated pathologic and pathogenetic features of VMC, BDA, BAF, and related lesions, with in depth discussion of malignancy and SD-iCCA. No systematic reviews have summarized these benign biliary lesions/neoplasms. Based on the comprehensive analysis of these three lesions referring to the malignancy and invasive carcinoma, we propose three categories of “biliary lesions/neoplasms arising in the hepatic parenchyma” (BLNP). (i) “Traditional BLNP,” which includes typical, benign-like VMC and BDA. BAF without cytological atypia, if it exists but was not reported, could be included in this category. (ii) “Unusual/dysplastic BLNP,” which is a constellation of unusual or dysplastic VMC and BDA, and BAF with dysplasia. Recently, peculiar iCCA with distinctive tubule-cystic pattern (TCC) or TCC with BAF-like lesions (AL-TCC) has been reported [35,38], different from conventional SD-iCCA, and SD-iCCA with ductal plate malformation pattern (dDPMP) [39,40,41] were reported, and both shared several overlapping pathologic features and showed invasive growth. These two diseases were categorized together as “low-grade malignant BLNP”. While the first category is benign and does not seem to be related to SD-iCCA, some of the second category and all of the third category may be neoplastic and malignant. It seems likely that SD-iCCA precursors may be included or identifiable in unusual/dysplastic BLNP, and that low-grade malignant BLNC may be a unique carcinoma associated with or followed by conventional SD-iCCA.

## 2. VMC and Related Lesions

**Figure 1:**
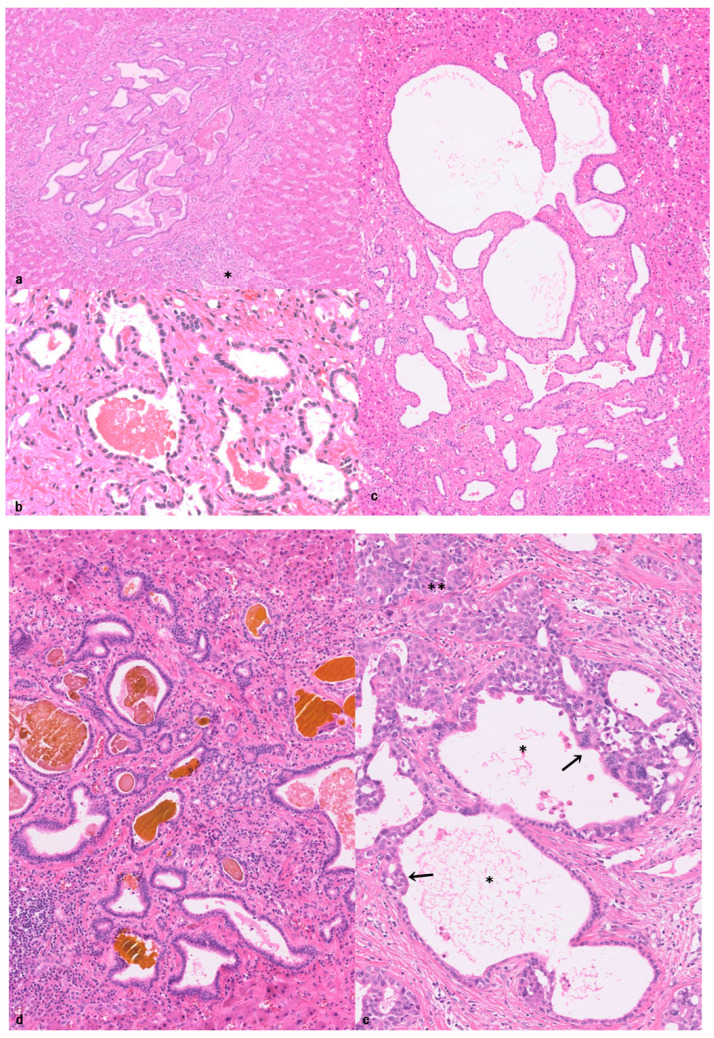
von Meyenburg complex. (**a**) The von Meyenburg complex shows a nodular lesion composed of irregularly shaped ductules embedded in fibrous stroma adjacent to the portal tract (*). Some of the ductules contain bile. Hematoxylin-Eosin (HE). (**b**) Lining epithelia of the von Meyenburg complex are cuboidal, and nuclei are regular and not hyperchromatic. HE. (**c**) One VMC shows variable-sized microcystic lesions and irregularly formed ducts lined by a single layer of cuboidal benign epithelia. Some of the ductules contain bile. HE. (**d**) The VMC shows a nodular lesion composed of irregularly formed small ducts with hyperchromatic nuclei and nuclear stratification embedded in fibrous stroma. Some ducts contain bile. HE. (**e**) The VMC (*) adjacent to invasive carcinoma (**) is lined by a single layer of benign biliary epithelia and also highly atypical epithelia, similar to surrounding invasive carcinoma (arrow) with an abrupt transition between them. HE.

VMC (Figure 1a–c), also called biliary hamartoma and bile duct hamartoma [10,27,29,30], belongs to the spectrum of ductal plate malformation (DPM) [30,42]. A majority of VMCs have been thought of as benign biliary lesions, but some VMCs could be related to the development of SD-iCCA [6,25,27,37].

### 2.1. DPM

#### 2.1.1. Definition

DPM may reflect the persistence of ductal plate during the fetal liver after birth due to abnormal arrest of ductal plate remodeling or refer to so-called “ductal plate-like structures” after birth [29,39,42,43]. DPM is characterized by the following gross and/or microscopic features [30,42,43]: (i) anastomosing tubular or cystic lumen lined by a single layered biliary epithelium, (ii) protrusion and balb-forming growth of the duct walls lined by a single-layered biliary epithelium into dilated tubular ductal or cystic bile duct lesions, and (iii) islands (bridge) of fibrous tissue lined by a single-layered biliary epithelial cells in dilated bile ductal or cystic lumen [43].

#### 2.1.2. Classification

Several non-neoplastic and neoplastic hepatobiliary diseases or lesions have been reported to exhibit the unique feature of DPM [19,39,42,43,44,45], and they can be subdivided into several categories (Table 3).

Non-neoplastic diseases include (i) congenital hepatic fibrosis (CHF) and (ii) Caroli’s disease [42,43], which show diffuse distribution in the liver, and (iii) VMC, which shows scattered and multiple distribution in the liver [10,27,42].

Neoplastic diseases include biliary fibroadenoma (BAF), iCCA with DPM pattern (DPMP), and adenofibroma-like tubulocystic cholangiocarcinoma (AL-TCC) [25,32,35,36,37,38,39,40,41,42,43,44,45,46].

Herein, we review and discuss VMC, referring to its neoplastic and malignant features. “VMC” is classified into three categories: (i) usual VMC, (ii) unusual/dysplastic VMC, and (iii) iCCA with DPMP. While CHF and Caroli’s disease are complicated by iCCA [47,48], these two diseases are well-established rare diseases and will not be discussed as a precursor lesion of usual SD-iCCA here.

### 2.2. Usual VMC

#### 2.2.1. Morphology, Incidence, and Clinical Features of Usual VMC

##### Morphology

Usual VMCs are usually small, well-circumscribed, uniform microscopical lesions (0.5 mm to 3 mm and <5 mm) scattered as small gray–white to green-tainted spots in the hepatic parenchyma of both hepatic lobes [10,25,26,27,29,44]. Some may be larger (>5 mm). Histologically, usual VMCs are located within the hepatic parenchyma adjacent to the portal tracts (Figure 1a). They are composed of irregularly shaped and variably dilated ductal and ductular lesions, variable microcystic changes, with branchings, lined by a benign single layer of low-cuboidal biliary epithelial cells with uniform cytological and regular nuclear appearance, embedded in fibro-collagenous stroma (Figure 1b,c). These ducts and ductules frequently contained bile-stained granular material or inspissated bile or proteinaceous fluid in their lumen, though some do not contain such bile or proteinous materials in the lumen. The VMC epithelial cells are immuno-positive for biliary cytokeratin (cytokeratin (CK)7 and CK19), CK8, and CK18. VMCs do not communicate with the bile duct lumen. Scattered non-neoplastic anomalous or hamartomatous bile duct clusters, which frequently coexist with usual VMCs, could be included in usual VMCs [30]. However, VMC-like lesions with atypical and neoplastic features are not included in usual VMCs.

##### Incidence and Clinical Features and Pathogenesis of Usual VMC

In autopsy livers without overt polycystic liver disease or primary liver neoplasms, VMCs were incidentally found in 3.1–5.6% of adults and 0–0.9% of children [30,44,45]. VMCs were detected clinically in 2.8% by routine ultrasound examination [49,50]. VMCs present a relatively benign disease course. While VMCs were generally multiple in the liver, their density was variable in individual cases: the majority with (i) few, smaller-sized VMCs (about 0.5–3 mm) were detectable in several liver sections from one case or (ii) several smaller-sized VMCs detectable in one liver section from one case [30]. (iii) VMCs were occasionally multiple, larger-sized (about 0.5–10 mm and more), and coalescent. (i)–(iii) may be related to each other.

A much lower incidence or absence of VMCs in children than in adults and more frequent VMCs in older people [30] suggest that such VMCs may develop in adults [27,30,44], and VMCs may occur secondary to inflammation, ischemia, or hepatocellular damage [27,30,44]. They are strongly associated with adult polycystic liver and kidney and may be considered part of the spectrum of this polycystic disease [29,42,51,52,53]. VMCs are also frequently associated with simple hepatic cysts.

While usual VMCs are occasional lesions (3.1–5.6%) in the general population [52], the incidence of iCCA is only 1.09 persons per 100,000 persons [1], showing a big discrepancy between the VMC prevalence of VMC and iCCA incidence in the general adult population, suggesting that usual VMCs are not causally related to the development of iCCA [26,37].

##### Differential Diagnosis

VMCs should be differentiated from BDA and BAF (Table 2) as well as bile ductular proliferation [27,28,54], peribiliary glands [10,55], and well-differentiated iCCA and metastatic adenocarcinoma [23,27,56,57].

### 2.3. Unusual/Dysplastic VMCs

#### 2.3.1. Unusual VMCs (VMCs in the Background Livers of Primary Hepatobiliary Neoplasms) and Chronic Liver Diseases

VMCs frequently found in the background livers of SD-iCCA and other primary liver cancers and also in chronic liver diseases were designated as “unusual VMCs” here [6,25,26,30,58].

##### Morphology and Clinical Features

VMCs were frequently detected in the background livers of SD-iCCA (24.3–34%) compared with LD-iCCA (12.8%) and with hilar CCA (17.6%) in adults [6,25,30], much higher than VMCs in non-neoplastic adult livers (3–5.2%) [26,31,51]. Interestingly, VMCs were also found in the background livers of 28% of hepatocellular carcinoma (HCC) and 33.3% of combined hepatocellular–cholangiocarcinoma (cHCC-CCA) [6,59,60], suggesting that VMCs are rather prevalent in the background livers of primary liver carcinomas. Aishima et al. reported VMC in 12 of 39 cases with chronic liver disease [58,61], though Jain et al. reported VMCs absent in 15 cases of cirrhosis without associated carcinomas [25].

##### Pathogenesis

Usual VMCs in general autopsied livers and unusual VMCs in the background livers of SD-iCCA showed similar histological and immunohistochemical phenotypes [30], suggesting that the former may not be different from the latter. However, an association of VMCs with primary hepatobiliary neoplasms, particularly SD-iCCA and chronic liver diseases, was not a mere coincidence but likely presented a phenomenon related to the occurrence or presence of primary liver carcinomas, particularly SD-iCCA and cHCC-CCA (a CCA component), and chronic liver diseases [6,25,30,44,58,61].

#### 2.3.2. Characteristics of iCCA with and Without VMCs

iCCA can present different clinicopathological features with reference to their co-existence with VMCs in the background liver [26]. That is, VMC-associated iCCAs are smaller in size and well to moderately differentiated with features of anastomosing glandular architecture, mimicking ductal carcinomas in situ (DCIS)-like growth pattern. They likely showed features of peritumoral lymphocytic infiltrate, complete pushing border, and central fibrous scar. In contrast, VMC-non-associated iCCA showed a predominant papillary, cribriform or solid growth pattern associated with aggressive features, such as tumor necrosis, perineural invasion, lymphovascular invasion, and positive surgical margin. These findings suggested that the developmental processes of VMC-associated iCCAs may be different from VMC non-associated iCCAs, suggesting that VMCs found in the background livers may be involved in unique cholangiocarcinogenesis different from ordinary cholangiocarcinogenesis without unusual VMCs.

#### 2.3.3. Dysplastic VMCs (VMCs with Atypical Features or In Situ Carcinomas)

##### Morphology and Clinical Features

VMCs exhibit occasionally atypical changes, such as nuclear swelling, hyperchromasia, or pseudostratification, and disordered polarity to a variable extent (dysplastic changes) (Figure 1d), and they are exclusively found in the background livers of iCCAs [25,31,37,62]. They can be bigger or coalescent. Jain et al. reported that dysplastic VMCs were observed in 19 of 41 (46%) patients with CCA and VMC [25], and they were characterized by architectural reminiscence of VMC and cytologic atypia blending in a continuum from benign to dysplasia/carcinoma in situ [25,37]. Our study [30] also showed that such dysplastic VMCs were found in 5 of 55 cases (9.1%) of SD-iCCAs in which dysplastic VMCs were found within the tumor or at the borders of the tumor, but not found in the liver remote from the tumor. Such VMCs were not found in the background liver of SD-iCCAs without VMCs and were not found in non-neoplastic autopsy livers [30].

##### Significance of Unusual/Dysplastic VMCs

Dysplastic VMC May Progress to iCCAs?

Several findings have suggested histologic progression from VMCs to intermediate lesions (dysplastic VMCs) to iCCAs [25,26,31,37,62]. The proximity and continuity of benign and dysplastic cells within the VMCs may raise the possibility of an ongoing transition from benign to dysplastic VMCs [25,26,37,62]. Such unusual/dysplastic VMCs may arise secondarily in adult livers that are prone to develop iCCA. It is possible that the putative stem or progenitor cells in the hepatic parenchyma regain the capabilities to develop ductal plate as a result of genetic alterations, and then, focal and scattered DPM features may develop as a form of VMC.

These changes are associated with progressively increasing Ki67 labeling index and high p53 expression [25]. A low p53 expression level was observed in the benign and low-grade dysplastic VMCs, and a high p53 expression level was observed in the high-grade dysplastic VMCs, suggesting that VMCs might progress with time, eventually leading to dysplastic transformation to iCCA [26,37,62]. P16^INK4a^ inactivation was observed in malignant parts of VMCs but not in their benign parts, which may be one of the underlying molecular mechanisms driving the malignant transformation in VMCs. In a study to evaluate allelic imbalance (loss of heterozygosity (LOH)) in VMC-associated iCCAs, iCCAs and coexisting VMCs exhibited loss of heterozygosity at 5–7 and 0–3 loci, respectively, supporting the neoplastic evolution of VMC [37,62].

Dysplastic VMCs May Reflect Cancerization?

Another process could be operative in the formation of dysplastic VMCs [30]. In CCAs and pancreatic duct adenocarcinomas, invasive carcinomas infiltrate again into the non-neoplastic epithelial layer of the pre-existing bile ducts and colonize which is called cancerization [63,64]. That is, cancerization can also occur in the pre-existing VMCs, resulting in the formation of dysplastic VMCs, particularly those within or at the border of the tumor [9,30]. That is, invasive carcinomas invade into the lumen of bile ducts and ductules of the pre-existing VMCs with an abrupt transition between pre-existing non-neoplastic biliary epithelia and cancerized epithelia (Figure 1e). In this context, such dysplastic VMCs may merely reflect secondary cancerization of pre-existing VMCs by iCCAs [30].

While some dysplastic VMCs may reflect precursor progression to iCCA, the others could be secondary cancerization. At the moment, objective markers to distinguish these dysplastic VMCs into either are not available, and this classification remains a future objective.

### 2.4. Well-Differentiated iCCAs Presenting a DPM Pattern (iCCAs with DPMP)

#### 2.4.1. Incidence, Clinical Features, and Morphology

##### Incidence and Clinical Features

iCCAs with DPMP affect adults of both sexes, and the background livers revealed frequently chronic liver diseases such as chronic B viral hepatitis. Chung et al. [19] reported that iCCAs with DPMP were found in 5 (2.9%) of 175 iCCAs. Patients with this subtype showed a favorable post-operative prognosis [19,30,65].

##### Morphology

**Figure 2:**
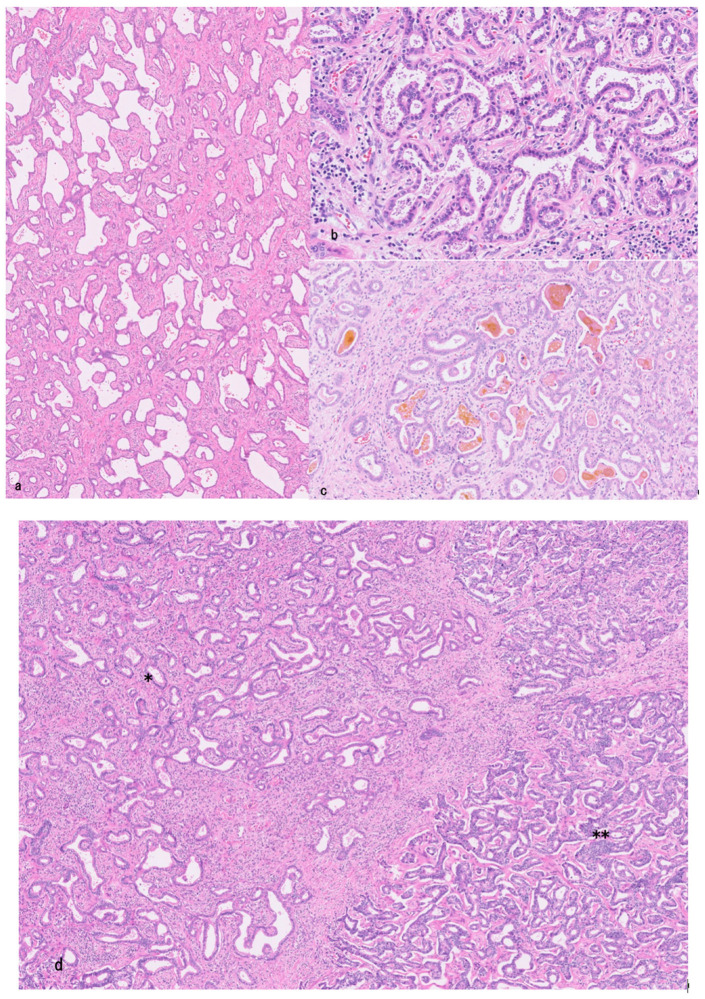
Small duct-type intrahepatic cholangiocarcinoma with ductal plate malformation pattern. (**a**) Well-differentiated cholangiocarcinoma showing irregularly shaped ductules embedded in fibrous stroma HE. (**b**) Higher magnification shows well-differentiated carcinoma with peculiar pattern sharing with ductal plate malformation. HE. (**c**) This part shows bile plugs in dilated lumen of well-differentiated carcinoma. HE. (**d**) Carcinoma showing ductal plate malformation (*) is adjacent to conventional cholangiocarcinoma (**). HE.

Grossly, the tumor is a single nodule (ranging from 1.5 to 6.6 cm in diameter) and is whitish and solid without a fibrous capsule. Microscopically, the tumor is composed of many vague, small nodular carcinomatous areas with desmoplastic reactions, and neoplastic glands had an irregularly dilated lumen lined with a single layer of cuboidal or low columnar carcinoma cells and irregular protrusions and bulges, resembling DPM (Figure 2a–c) [19,30,39,40].

#### 2.4.2. Progression of iCCAs with DPMP to SD-iCCAs and Relation to Other iCCAs

While the histogenesis of iCCAs with DPMP remains speculative, this type can present as (i) invasive DPMP alone [30,39], (ii) invasive DPMPs with conventional SD-iCCAs (Figure 2d) [30,39], and (iii) DPMPs with combined HCC-CCAs [46,66,67], when diagnosed. In (i) and (ii), DPMP components were predominant. Invasive DPMPs alone are composed of benign-looking biliary epithelia and share the features of DPMs including VMCs [30,39]. At its border, the carcinoma of DPMP types in (i) and (ii) shows replacing growth against the surrounding parenchyma (non-neoplastic hepatic lobules or regenerative nodules), and intact portal tracts are frequently incorporated within the tumor. These findings suggest that iCCA with DPMP might have arisen from dysplastic VMCs and might have been associated with SD-iCCA [30]. Differentiation of iCCAs with DPMP from larger dysplastic VMCs may be occasionally subjective, suggesting a transition of two neoplastic lesions.

As will be discussed later, SD-iCCAs with DPMP were recently reported to share overlapping features of TCC with BAF features [35,38].

#### 2.4.3. Molecular/Genetic Alterations in iCCAs with DPMP

iCCAs with DPMP were negative for mucin, p53 was scarcely expressed, and the Ki-67 labeling index was <10%; they were frequently positive for CK19 and epithelial membrane antigen (EMA), and variably stained for neural cell adhesion molecules. This type of iCCAs also showed unique genetic alterations, such as mutations in *BAP1*, *CDKN2A*, and *ARID1A* with *FGFR2* fusion [9,19,41,46].

## 3. Bile Duct Adenomas and Related Lesions

### 3.1. Bile Duct Adenomas

Bile duct adenoma (BDA) is a benign epithelial lesion composed of monotonous proliferation of small bile ducts or bile ductules, presenting a small nodular growth [6,27,59,60,68,69]. BDA could be pathogenetically heterogeneous [27], and the majority might be a benign proliferative lesion [1,27], while some BDAs could be a true neoplasm and be a candidate for precursor lesions of SD-iCCAs [69,70,71,72,73].

#### 3.1.1. Incidence and Clinical Features

BDAs are found in 0.6% of large series of autopsies to 2.4% of large series of surgical resection cohorts [6,60,68,74,75]. They are mostly identified in adults, affecting both sexes. Clinically, BDAs are detected incidentally in diagnostic images for other purposes or during intra-abdominal surgery or at autopsy [23,68,76]. BDAs are typically solitary (83–90%) and mainly found in the subcapsular region (90%) of the normal liver (Figure 2a) [1,27,68]. The follow-up of the surgically treated patients confirmed the benign feature of BDA [77].

#### 3.1.2. Morphology

##### Gross and Histology

**Figure 3:**
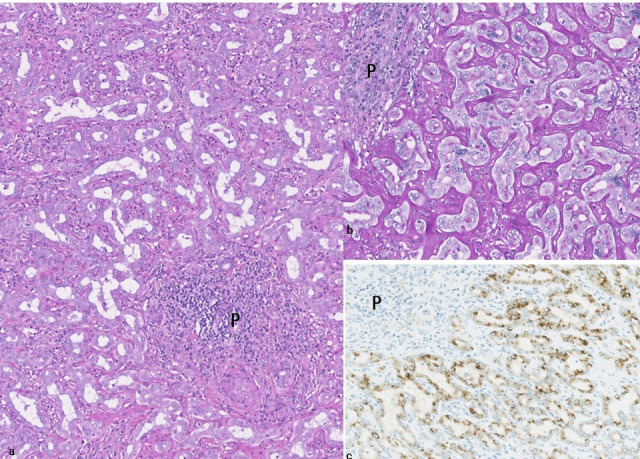

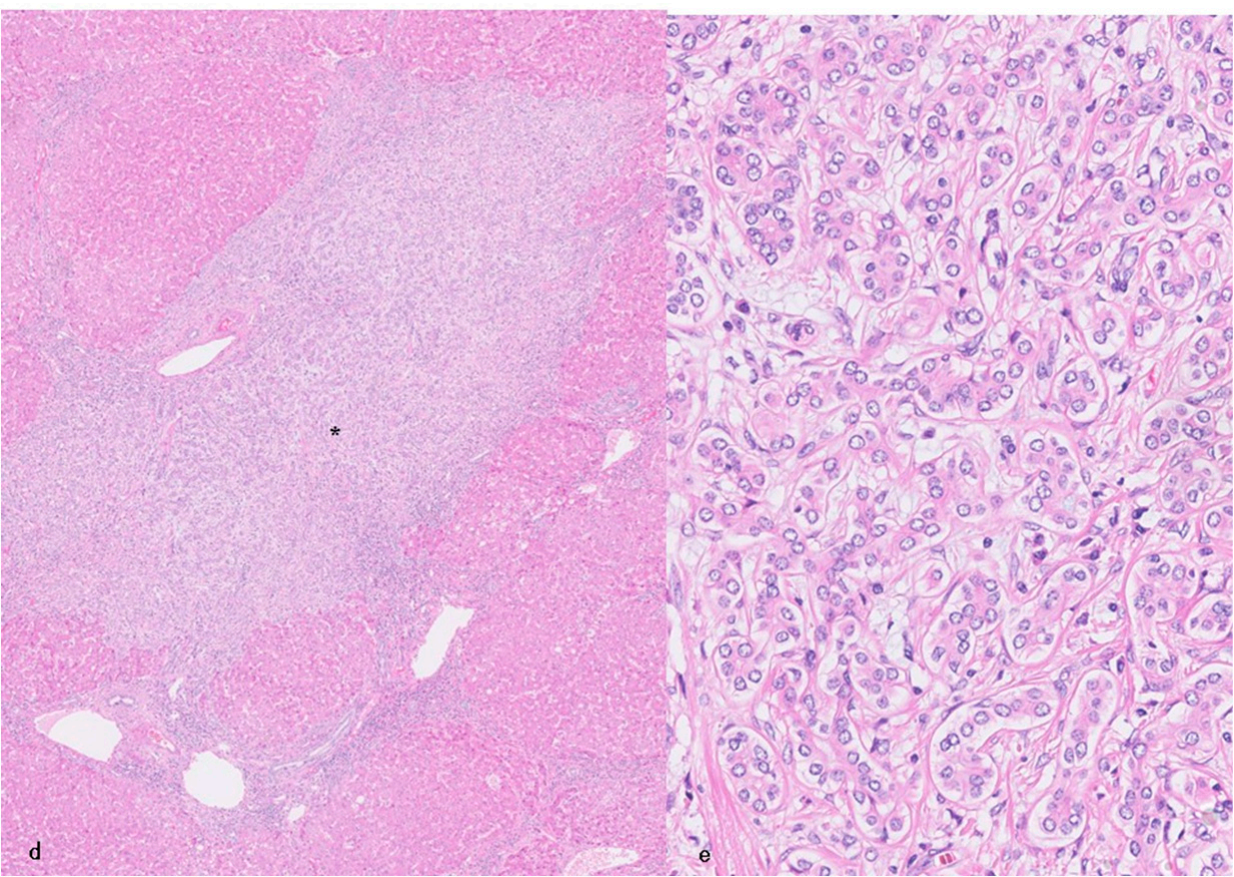
Bile duct adenoma. (**a**) This lesion is composed of monotonous small ducts with fibrous and inflammatory stroma. One smaller portal tract with inflammatory cell infiltration (P) is incorporated in the tumor. HE. (**b**) d-PAS-positive mucinous materials are found on the luminal side of bile ducts. P, portal tract. PAS after diastase digestion (d-PAS). (**c**) These smll ducts constituting the tumor are positive for MUC5AC. P, portal tract. Immunostaining for MUC5AC and hematoxylin. (**d**) Bile duct adenoma (*) located in deep hepatic parenchyma. The background liver shows chronic hepatitis approaching liver cirrhosis. (**e**) The tumor is composed of compacted smaller ducts with a slit-like lumen and with few stroma. HE.

BDAs are well-circumscribed, nonencapsulated, whitish or tan-colored solid lesions [1,27,68]. In the largest reported series, BDAs were usually less than 1 cm (90%) and ranged from 1 mm to 20 mm (mean, 5.8 mm). BDA presents a patternless proliferation of uniformly shaped and sized bile ductules or small interlobular bile ducts lined by single-layered cuboidal epithelia with small and regular nuclei and an intact basement membrane and with a small or little lumen (Figure 3a), and ductal and ductular proliferation is more frequent in the margin. There are usually no cellular and nuclear atypia suggestive of dysplasia or carcinoma. BDAs are embedded in a connective tissue stroma showing varying degrees of chronic inflammation and fibrosis, and the percentage of fibrosis with inflammatory cell infiltration including nodular lymphoid aggregates is higher at the margin. Normal portal tracts are usually incorporated within the lesion, particularly at the peripheral area. BDAs lack infiltrative growth and the margins can be seen to push against the hepatic parenchyma. The background liver usually shows no evidence of chronic advanced liver diseases including cirrhosis.

Mitotic activity is inconspicuous, and Ki-67 labeling index (LI) is low (average 2%, never >10%) in the ductal and ductular epithelia [56,78], suggesting that they have limited growth potential. p16^INK4a^ was expressed in approximately 80% of BDAs, and p53 and AFP expression were negative [59,60].

##### Mucin Histochemistry and Immunohistochemistry

Alcian blue or mucicarmine positive acidic-type mucin is usually positive in BDAs in the supranuclear cytoplasm and at the luminal border of ductal or ductular epithelia, to various extents (Figure 3b) [27,68]. Biliary cytokeratin (CK7 and CK19), CK8, and CK18 are diffusely positive, and CD56 is focally expressed in BDA [57,76]. CK20 is negative in BDAs [27,57,59,60]. MUC6 (94%), MUC5AC (90%), TFF2 (80%), and foregut antigens D10 and 1F6 are frequently positive in BDAs, to various extents (Figure 3c) [59,76].

#### 3.1.3. Differential Diagnosis

Differentiation of BDA from well-differentiated iCCA including CoCC or metastatic carcinoma is very important but still controversial [23,27,56,57,79,80] (Table 2). This differentiation can be made on the basis of tumor size, their cellular atypia, hyperchromatic nuclei, and invasive features [68]. The Ki-67-LI was low (average 2%, never >10%) and strong p53 staining was not seen in BDA [56,81]. p16 is expressed in BDAs but lost in carcinomas. Cholangiolocellular carcinoma (CoCC) and SD-iCCA express MUC1 but fail to express MUC 6 and acidic-type mucin in their cytoplasm [82], thus different from BDA [83]. EZH2 and IMP3 expressed in SD-iCCA are not expressed in BDAs (Table 4 and Table 5) [59,60,75]. In addition, genetic alterations of *p53*, *ARID1A*, *PBRM1*, *MTAP*, *IDH1*, *KRAS*, and *TERT promoter* are detected in most SD-iCCA, even small-sized ones but absent in BDAs [60].

#### 3.1.4. Pathogenesis

BDA may encompass a heterogeneous group of lesions with similar morphological appearance but with variable pathogenesis ranging from reactive proliferation to true neoplasm [6,61,73] and some may have the potential to progress to biliary malignancy [74].

##### Reactive Changes

BDA is generally and currently regarded as a reactive process to hepatic parenchymal injury. That is, a trigger of trauma or inflammation or ischemia may result in a reactive focal bile ductular proliferation in a tumor-like lesion [68,73], because of their smaller size, preferential subcapsular location, and limited growth potential [1,27,60,68,73].

##### Peribiliary Gland Hamartoma

The regular cell and nuclear pattern of BDA resembles peribiliary glands in normal livers [10,55,76]. That is, the small ducts of BDA consisted of mucous cells expressing MUC6 and MUC5AC and two foregut antigens (D10 and 1F), similar to the peribiliary glands, suggesting an origin of BDA from peribiliary glands [10,61,76,83]. Actually, BDAs located around a large-caliber bile duct where peribiliary glands are physiologically located were reported [76,84]. So, Bhathal et al. proposed BDAs to be called “peribiliary gland hamartomas” [76]. However, Hughes et al. proposed that BDAs rather resembled inflamed peribiliary glands and may develop as a localized biliary healing response along with pyloric gland metaplasia/peribiliary glandular differentiation, which better supported a response to injury [84].

##### Neoplasm

The following findings support a neoplastic character of BDA and suggest its possible relation to biliary malignancy, particularly SD-iCCA. Somatic *BRAF p.V600E* mutation was reported in 53–87.5% of BDAs by using molecular/genetic approaches [61,69,70,74], suggesting that mutated BDAs with *BRAF* mutations may be a precursor for the subset of iCCA harboring *BRAF* mutations (3–5% of iCCA) [58,85,86,87,88,89]. Interestingly, some BDA with the *BRAF p.V600E* mutation exhibited unusual features, such as multiplicity [85], larger size and microcystic change, deeper location in the liver parenchyma, associations with SD-iCCA, and chronic advanced liver diseases [69]. Multiple *BRAF V600E*-mutated BDAs which were reported in chronic liver disease may be designated as “*BRAF*-associated bile duct adenomatosis [85]”. However, multiple BDAs without the *BRAF V600E* mutation were also reported [61], and they may harbor different molecular/genetic alterations from *BRAF* mutations [85].

Recently, Sasaki et al. reported that those with *BRAF* mutations were not detected in 26 BDAs from Japanese cohorts by molecular/genetic approaches [60]. Most studies which detected the *BRAF*-V600E mutation in BDA were conducted in Europe [61,69,70,74,85], so the diagnostic criteria for BDA and the background livers of BDAs may not be consistent across these studies. Therefore, unknown geographical and environmental differences may be related to the different frequencies in the *BRAF-p.V600E* mutation in BDAs in different geographical regions.

### 3.2. Relation of BDA to SD-iCCA

#### 3.2.1. Unusual/Dysplastic BDAs

In addition to above-mentioned BDAs, several unusual or atypical features were reported in BDA (Table 6) [6,58,59,60,61,69,72,74,75,90,91,92,93], such as multiple BDAs (bile duct adenomatosis), deeper location in the hepatic parenchyma (Figure 3d,e), BDAs in the background livers of SD-iCCAs and chronic advanced liver diseases, and BDAs of large size and with compact growth and few fibrous stroma. Mucin-negative BDAs could also be regarded as unusual BDAs.

##### BDAs Frequently Found in the Background Livers of SD-iCCA and Chronic Liver Diseases

Sasaki et al. reported that the prevalence of BDAs was significantly higher in the background livers of SD-iCCAs compared with control livers and other primary liver carcinomas [59]. That is, BDAs were found in the background livers of 35.7% of SD-iCCA cases [6,59,60,75], higher than those (0.5–2.0%) in a large-series of BDAs in general population, those (4.9%) in cHCC-CCA, and those (10%) in HCC [59,68,69], suggesting that these frequent BDAs may be primarily or secondarily related to the development of SD-iCCA and possible adenoma–carcinoma sequence [59,91]. Aishima et al. also reported that 35 BDAs were observed in 39 cases with chronic liver disease [58]. Sasaki et al. reported that while genetic alterations such as *p53* and *ARID1A* were detectable in most SD-iCCAs, even small-sized ones, as mentioned above, these genetic alterations were not detected in these BDAs [60], suggesting that another genetic hit may be responsible for the development of carcinoma in BDAs.

##### BDA with the BRAF p.V600E Mutation

BDAs harboring the *BRAF p.V600E* mutation may be a true neoplasm and could progress to carcinoma sequence, and in this context, these BDAs may be a precursor lesion of SD-iCCAs [61,68,69,70,74]. In a previous report, the *BRAF p.V600E* mutation was detected in BDAs and iCCAs in 2 out of 4 iCCAs associated with BDAs present in the same liver, suggesting that iCCAs with the *BRAF p.V600E* mutation may have arisen from mutated BDAs, and such BDAs may represent a precursor lesion to the subset of iCCA harboring the *BRAF V600E* mutation (3% of iCCA) [70].

##### BDA in AAT Deficiency

Patients with homozygous and heterozygous α_1_-antitrypsin (AAT) deficiency are at a risk of developing iCCA in addition to HCC, and cHCC-CCA [27,74,93]. BDAs have been occasionally reported in AAT deficiency, and such BDAs may be a result of neoplastic transformation of biliary epithelia triggered by the accumulation of AAT globules [94]. Angkathunyakuk et al. [74] reported that eleven biliary lesions from five patients with AAT deficiency (six BDAs from three cirrhotic patients and three BDAs and two ICCs from two non-cirrhotic patients) were identified, and most BDAs in AAT deficiency expressed hepatic progenitor cell (HPC) related immunohistochemical profile such as CD56, EpCAM, CD133, and CA19-9, and *BRAF V600E* mutation was detected in 87.5% of such BDAs. The frequent *BRAF V600E* mutations in BDAs in patients with AAT deficiency may support the neoplastic nature of BDAs, but not necessarily their progression to iCCAs [74].

##### Dysplastic BDA

BDAs with the *BRAF p.V600E* mutation or those associated with SD-iCCAs showed occasionally mild dysplastic changes and they could be included in dysplastic BDA, and some unusual BDA had features of VMCs [23,61,69,70,73,77,83].

#### 3.2.2. Malignant Transformation of BDA to iCCA

Malignant transformation of BDA has been rarely reported, suggesting an adenoma–carcinoma sequence [71,73,91].

As mentioned above, BDA was frequently identifiable in the background liver of SD-iCCA, and some BDA presented dysplastic changes and *BRAFp.V600E* mutation, suggesting that some BDA could be a precursor of SD-iCCA. However, as mentioned above, BDA itself could be heterogeneous and the diagnostic criteria of BDA itself were not unified internationally, so the exact progression processes of BDA to SD-iCCA remain to be clarified.

### 3.3. Variants

Some variants of biliary neoplasms are named as BDAs but are different from typical BDA [85,93,95,96,97,98,99]. These variants could already be malignant and may not be parts of the BDA spectrum.

#### 3.3.1. Clear Cell Adenoma

Clear cell BDAs mimicking metastatic renal cell carcinomas have also rarely been reported and have been interpreted as atypical BDA [85,95]. These variants could not be as part of the spectrum of usual BDAs with frequent expression of gastric phenotypes including acidic mucin.

#### 3.3.2. Oncocytic Adenoma

BDAs totally composed of oncocytic cells have rarely been reported. The majority of so-called oncocytic neoplasms of other organs are generally malignant [95,96], so this oncocytic variant is also malignant. One case of an oncocytic variant with abundant mitochondria and an NRAS mutation was recently reported [86].

#### 3.3.3. BDA with Neuroendocrine Markers

BDAs with neuroendocrine components have also been reported [100].

## 4. Biliary Adenofibroma (BAF) and Related Diseases

BAF has been classified as a benign biliary neoplasm [32,35,101,102,103,104,105,106,107,108], though BAF is also generally thought to have potential for malignant progression [9,32,34,35]. More than half of the resected BAF had an area of overt adenocarcinoma [102].

### 4.1. BAF

#### 4.1.1. Incidence and Clinical Features of BAF

BAF is an extremely rare primary biliary neoplasm affecting adults with a slight female predominance [32,33,34]. The clinical outcomes suggest that the neoplasms are only slowly progressive. While the course appears benign, malignant transformation may supervene if the lesion is left untreated. Most patients (81.4%) were alive and disease-free after resection with occasional relapse (14%) or death of disease (4.6%) [102]. Its extreme rarity, coupled with an incompletely understood histogenesis, may lead to misclassification with other similar entities such as multicystic biliary hamartoma (MCBH) [34].

#### 4.1.2. Pathology of BAF

**Figure 4:**
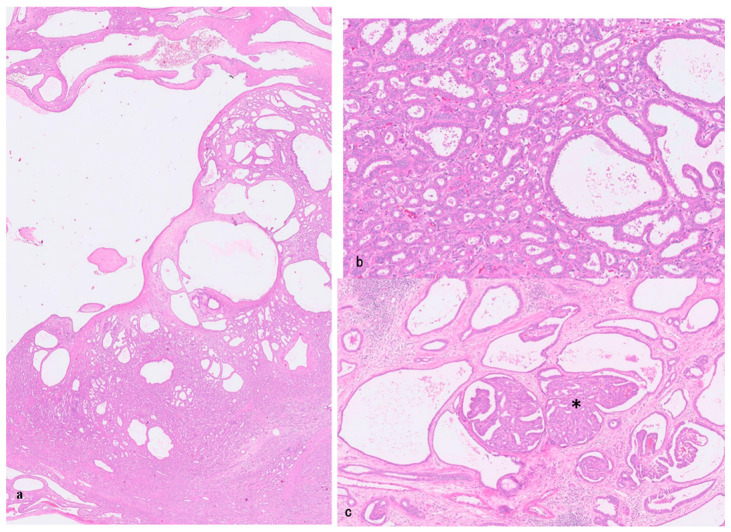
Biliary adenofibroma. (**a**) Cystic-microcystic lesions with solid and tubular components. HE. (**b**) Well-differentiated tubular components are found. HE. (**c**) The cysts of various sizes are seen, and some show cystic complex papillary proliferation and cribriform formation (*). HE.

The tumor is a mass-forming (MF) tumor, and is typically solitary and 1.7–16 cm in diameter (Table 2) [1,32,33,102,109]. It is non-encapsulated, well-circumscribed, and round to oval, and it usually shows mixed features, ranging from cysts intermingled with solid nodules to spongy, non-capsulated lesions [32,101,109]. BAF typically presents histologically as a low-grade, complex, microcystic and tubuloglandular bile duct structure, resembling VMC or DPM (Figure 4a,b) [1,32]. The cysts are of various sizes, and intracystic complex papillary proliferation and cribriform formation are histologic components of BAF (Figure 4c) [1,32]. These structures are lined by low columnar to cuboidal non-mucin-producing biliary epithelium [1,32,33]. The lining cells typically have amphophilic cytoplasm, bland nuclei with inconspicuous nucleoli, and minimal contour irregularity. The background stroma is collagenous and contains bland myofibroblasts. The majority or all of BAF show mild to marked cytologic atypia, compatible with its neoplastic character [33,34,102]. BAFs with invasive components accounted for 52.7% of the literature [102].

The epithelial lining presents immunoreactivities of biliary phenotype, such as EMA, CK7, CK19, and CA19-9 [32,34] as seen in small bile ducts and bile ductules [11]. However, positive expression of D10 and no expression of IF6 of BAF were similar to VMC [102]. The Ki-67 proliferation index is never >10% in the epithelial component and is always <1% in the stromal part.

#### 4.1.3. Differential Diagnosis

BAF should be differentiated from the following diseases/lesions in addition to VMC, BDA, well-differentiated cholangiocarcinoma, and bile ductular proliferation (Table 2).

##### MCBH

MCBH exhibits an intrahepatic, partly cystic mass lesion, histologically characterized by cystically dilated, large ducts embedded in fibrous connective tissue [32,34]. Unlike BAF, peribiliary glands can be present in the tumor [34]. A diagnostic clue is the presence of unremarkable portal tracts within the tumor.

##### Complex Papillary Cribriform Pattern

Complex papillary cribriform pattern (CPCP) resembling the intracystic complex papillary cribriform pattern of BAF is occasionally and variably found in conventional SD-iCCA (Nakanuma Y et al., in submission), though CPCP showed more atypical and irregular features such as nuclear atypia and stratification deserved to be called carcinoma.

#### 4.1.4. Pathogenesis of BAF

Its expansile growth, possession of mitoses, and foci of epithelial tufting and cellular atypia favor a neoplastic process. BAF resembles VCM or DPM, so there could be similarities between BAF and other neoplasms with DPM features [32,35]. Chua et al. reported two cases of iCCA associated with fibrocystic liver disease with DPM and BFA, suggesting the linkage between DPM and BAF [109].

#### 4.1.5. Molecular and Genetic Alterations

Mattiolo et al. reviewed 55 cases of BAF with molecular investigations demonstrating genetic alterations typically seen in SD-iCCA, including mutation of *RID1A*, *BAP1*, *PBRM1,* and *P53*, and *FGFR2* fusion [102]. Three tumors tested by array comparative genomic hybridization showed chromosomal copy number alterations, including amplifications of *CCND1* and *ERBB2* [108].

### 4.2. Relation of BAF to Malignancy and SD- iCCA

All or almost all BAFs present mild to marked cytoarchitectural atypical changes. In addition, a considerable proportion of reported cases had areas of overt adenocarcinoma of SD-iCCA (37–50%) indicating a higher risk of malignant transformation of BAF [34,102,110]. BAFs were frequently associated with invasive components or iCCA [102].

#### 4.2.1. BAF with High-Grade Dysplasia and In Situ Carcinoma

A range of premalignant changes leading to invasive carcinoma is noted in about half of the reported cases with epithelial dysplasia including elongated hyperchromatic nuclei and nuclear pseudostratification, as well as architectural disturbance with intracystic complex papillary proliferation and cribriform formation [1,36,106]. The diagnosis of malignant transformation of BAF generally requires compelling histologic evidence of a true BAF, an abrupt transition between BAF and adjacent conventional SD-iCCA (Figure 3d), or the presence of high-grade dysplasia within BAF [36,103,104,105,106].

#### 4.2.2. iCCA Derived from and/or Related to BAF

Several types of carcinomas are reported to arise in or be associated with BAF, or to show BAF-like features [23].

##### Conventional SD-iCCA

Conventional SD-iCCA is most common [23,101,102,103,110].

##### Peculiar CCA with Distinctive Tubulocystic Pattern and Underlying BAF-Like Pattern

Recently, tubulocystic cholangiocarcinoma (TCC) [35] and adenofibroma-like tubulocystic carcinoma (AL-TCC) [38] were newly reported for this peculiar CCA, separately, though these two entities appear to be similar or are much overlapped, and so, “AL-TCC” was therefore adopted in this review [35,38]. This subtype is very rare and shows invasive growth, though the post-operative course is relatively favorable compared with conventional SD-iCCA [35,38].

**Figure 5:**
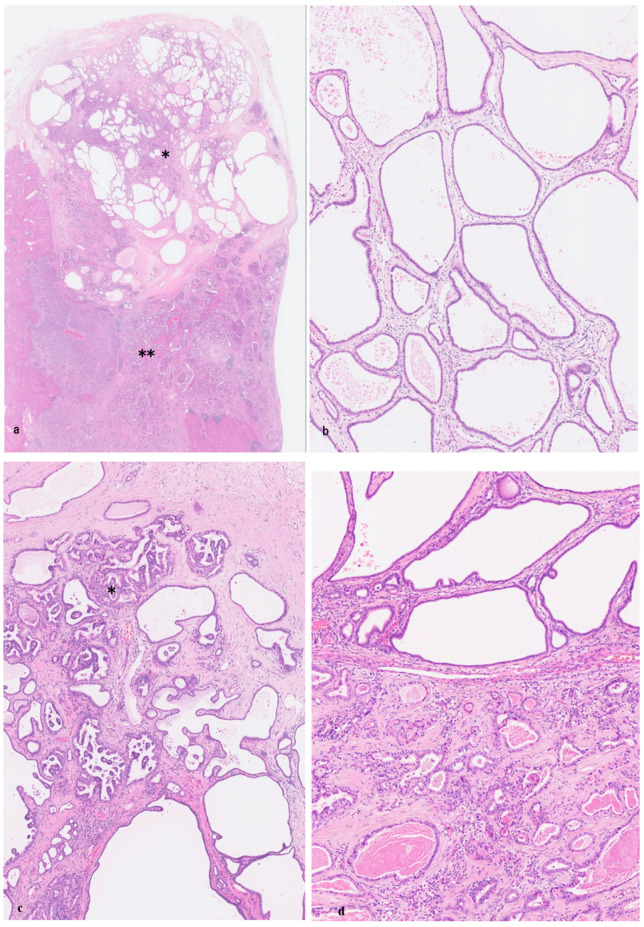
Adenofibroma-like-tubulocystic cholangiocarcinoma (AL-TCC). (**a**) This type of tumor is composed of cystic areas (*) resembling biliary adenofibroma and invasive carcinoma (**). HE. (**b**) Tubulocystic carcinoma showing well differentiation with fibrous stroma. HE. (**c**) Some of the cystic areas show cystic complex papillary proliferation and cribriform formation (*) as seen in biliary adenofibroma (Figure 4c). HE. (**d**) Cystic tubular neoplastic area is adjacent to invasive conventional cholangiocarcinoma (lower half). HE.

AL-TCC affects adults of both sexes. The mean size is 4.4 cm–5.5 cm. On cut section, both solid and microcystic areas appear in varying proportions, with the latter sponge-like areas appearing in varying proportions (Figure 5a). AL-TCC shows histologically a peculiar and cytologically bland tubulocystic pattern that closely resembles low-grade tubulocystic-type kidney cancers, including back-to-back microcystic units, and appears to have arisen in the background of BAF-like lesions [35]. TCC is deceptively benign-appearing tubulocystic glands, resembling BAF (Figure 5b), transitioning into more complex papillary lesion (Figure 5c) and conventional SD-iCCA (Figure 5d). AL-TCC may be less aggressive compared to conventional SD-iCCA. Foci of CoCC resembling BDA or DPMP similar or identical to iCCA with DPMP were also found [34].

Next-generation sequencing showed recurrent mutations in chromatin remodeling genes, such as *ARID1A*, *BAP1*, and *PBRM*1, and the actionable *FGFR2-MCU* fusion gene, in AL-TCC [35,38].

In addition to AL-TCC, BAF associated with conventional SD-iCCA and some BAF cases with malignant transformation may harbor the above-mentioned features of AL-TCC, so the re-reviewing of BAF with conventional CCA or BAF with malignant transformation is mandatory from the standpoint of the newly proposed disease concept of “AL-TCC”.

#### 4.2.3. Relation to iCCA with DPMP

iCCA with DPMP is an established variant of SD-iCCA, and its relation to BAF and AL-TCC will be discussed later.

## 5. Categorization of “Biliary Lesions/Neoplasms of Hepatic Parenchyma (BLNP)” into Three Groups with Reference to Their Neoplastic and Malignant Features

Biliary lesions/neoplasms are largely dividable into two categories with respect to iCCA [1,6,7,8]: (i) those arising in the lumen and peribiliary glands of the grossly visible bile ducts resulting in LD-iCCA and their precursors such as high-grade BilIN and IPNB [1,7,8], and (ii) those arising in the hepatic parenchyma resulting in SD-iCCA [6,9]. It is likely that the precursors and early malignant lesions of SD-iCCA could be identifiable in BLNP. As discussed above, VMC, BDA, BAF, and related lesions, all of which present biliary phenotypes of small interlobular bile ducts and bile ductules and are located in the hepatic parenchyma [6,9,11], could be comprehensively divided into the following three categories (Table 7). VMC and BDA in normal livers and BAF without cytological atypia, if it exists but has not been reported, could be categorized as “traditional BLNP”. Unusual/dysplastic VMC and BDA, BDA with the *BRAF V6001* mutation, and BAF with dysplastic changes or foci of in situ carcinoma, all of which are different from traditional BLNP and lack invasive growth but show overlapping or reminiscent features of traditional BLNP, could be categorized as “unusual/dysplastic BLNP”. SD-iCCA with DPMP and AL-TCC, which already show invasive growth but are different from conventional SD-iCCA, commonly show a favorable post-operative prognosis compared to conventional SD-iCCA [19,35,38,39,40]. So, these two malignant diseases could be categorized as “low-grade malignant BLNP”.

However, these three categories were proposed to function as a spectrum or constellation of hepatic parenchymal lesions reflecting neoplastic or malignant changes or reactive changes but not as a discrete class. Particularly, unusual/dysplastic BLNP was composed of BLNP with “unusual” or “dysplastic” features. That is, while some unusual/dysplastic BLNP could be neoplastic, the others could be simply: “typical” or “unusual” BLNP compared to traditional BLNP and not neoplastic. So, the features characterizing unusual/dysplastic BLNP do not always reflect progression risk. At the moment, there are no objective markers applicable to distinguish between them. Similarly, the boundary between unusual/dysplastic BLNP and low-grade malignant BLNP could be overlapping, and again.

### 5.1. Traditional BLNP

Usual VMC and BDA, which belong to this category, are distinguishable histologically from each other, and have been regarded as reactive proliferative changes or remnant developmental anomalies [27,30]. Wen et al. reported by using DNA flow cytometry that most BDA and VMC in which aneuploidy was not detected are benign entities and may not represent precursors to iCCA in which aneuploidy was detected in 47% [79]. The majority or all of the BAF reported so far is associated with mild to marked cytological atypia, suggestive of neoplastic character. If BAF without cytological atypia could exist, such cases may belong to this category but may present considerable overlap with and be difficult to differentiate from larger VMC.

### 5.2. Unusual/Dysplastic BLNP

Unusual/dysplastic VMC and BDA, BDA with the *BRAF p.V600E* mutation, and BAF with dysplastic changes, all of which are different from traditional BLNP, could constitute this category. Two or more of such lesions could be found in the same liver [58]. These lesions do not show invasive growth but could be associated with SD-iCCA at a variable frequency.

#### 5.2.1. Unusual/Dysplastic VMC and BDA and Dysplastic BAF

##### Frequently Detectable VMC and BDA in the Background Livers of SD-iCCA and Chronic Liver Diseases

BDA and VMC are relatively frequent in the background livers of 5.1–35.7% and 24.3–39.3% of cases of SD-iCCA, respectively [6,22,25,26,44,58,59,60], compared with a large series of control autopsy or resection cases [30,68]. These lesions were found adjacent to or within the tumor of SD-iCCA as well as away from it [6,59,60]. BDA and VMC are also relatively frequent in chronic liver diseases compared with controls [2,25,26]. Aishima et al. also reported that 35 BDAs and 12 VMCs were observed in 39 cases with chronic liver diseases [58].

##### Other Unusual Features of BDA (Table 6)

Other unusual BDAs such as those located in the deeper parenchymal parts, showing multiplicity and mucin-negative BDA [6], could be included in this category.

##### BDA with the *BRAF p.V600E* Mutation

Somatic *BRAF p.V600E* mutations were reported in 53–87.5% of BDAs [61,69,70,74] and such mutated BDAs with *BRAF* mutations may be important precursors for the subset of iCCA harboring *BRAF* mutations [61,70,89].

##### Dysplastic VMC, BDA, and BAF

Interestingly, some VMCs adjacent to the tumor and within SD-iCCA show dysplastic changes [6,22,25,26] and VMC found in the background liver of about 40% of SD-iCCA presented dysplastic features [25]. BDA with the *BRAFp.V600E* mutations exhibit cytological and nuclear atypia and/or other unusual features, such as multiplicity, larger size and microcystic change [69,70], deeper location in the liver parenchyma, and association with SD-iCCA and/or chronic advanced liver diseases. BAF with mild to marked dysplastic changes or foci of in situ carcinoma are also included in this category.

#### 5.2.2. Coexistence and Overlapping Features Among Unusual/Dysplastic BLNP

In the background liver of SD-iCCA and in chronic liver diseases, unusual VMCs and BDAs coexist occasionally [6,59,60,111], and some focal biliary lesions show mixed features of BDA and VMC or DPMP [25,70,77,93].

#### 5.2.3. Pathogenesis of Unusual/Dysplastic BLNP

The above-mentioned findings may raise a possibility that these unusual/dysplastic VMCs and BDAs may be closely related to each other, could have originated from one or a few cell lineages, and may have differentiated into different features. Some might have progressed to present with BDA traits, some with VMC traits, some with mixed or combined traits of BDA and VMC or DPM, suggesting that occurrence of such unusual/dysplastic VMCs or BDAs is a risk of or could be involved in the development of SD-iCCA. Actually, Hasebe et al. reported one case of iCCA arising in BDA with atypical epithelia and VMCs in the same liver, suggesting an adenoma–carcinoma sequence [91]. Based on these findings, unusual/dysplastic VMCs and BDAs could be different in pathogenesis and biological significance from traditional VMC or BDA from the beginning [6]. Dysplastic BFAs with features of VMCs and BDAs might have also undergone common pathogenesis as seen in other unusual/dysplastic BLNPs and have become a larger tumor. However, cancerization of traditional VMCs by SD-iCCA may also occur secondarily and result in the development of so-called unusual/dysplastic VMCs [30].

### 5.3. Low-Grade Malignant BLNPs

SD-iCCA with DPMP and AL-TCC could belong to low-grade malignant BLNPs [35,38,39,40,41]. Recently, Liao et al. raised a hypothesis that AL-TCC and iCCA-DPM form a common tumorigenic spectrum [38]. BAF associated with the component of conventional SD-iCCA may also be included in this category. Interestingly, comparison between AL-TCC and iCCA with DPMP revealed no significant difference in age, sex, tumor size, focality, lymphovascular invasion, and perineural invasion and outcome [35]. When AL-TCC and SD-iCCA with DPMP were grouped together and compared with conventional SD-iCCA, the combined cohort showed a stronger association with VMC, biliary cysts, and/or BDA (41%), less perineural invasion, ARID1A loss (65%), and a better patient outcome. Kaplan–Meier analysis revealed that ARID1A loss significantly improved patient survival, suggesting that AL-TCC and iCCA with DPMP may share clinicopathological and histogenetic characteristics, suggesting that both are closely related to each other and likely present a common continuum of tumorigenesis, different from conventional SD-iCCA [35].

### 5.4. Dysplastic/Unusual BLNC Could Be a Precursor of SD-iCCA and Be Related to Low-Grade Malignant BLNP

#### 5.4.1. Dysplastic/Unusual BLNC Could Be a Precursor of SD-iCCA

While traditional BLNPs are unlikely to be followed by conventional SD-iCCAs, unusual/dysplastic BLNP, particularly VMC and BDA, may represent SD-iCCA precursors and support precursor–carcinoma sequences. For example, unusual/dysplastic BLNPs are associated with conventional SD-iCCAs to a variable frequency [6,25,59,60,91] and unusual/dysplastic VMC/BDA are occasionally observed near or at the peripheral rim of conventional SD-iCCA [22,25,26,39]. Dysplastic VMC showed in situ-like lesions [25,26,31,62], and genetic and molecular alterations suggesting neoplastic changes were reported in dysplastic VMC [37,62]. BDAs were also shown to present *p.V600E* mutation frequently, and such mutation was actually identifiable in occasional cases of SD-iCCA [61,69,70,74]. It is likely that such unusual/dysplastic BLNPs might have been involved in the development of conventional SD-iCCAs, suggesting the malignant transformation of these BLNPs. However, no large-scale epidemiologic data are available to support the causal relationship between these unusual/dysplastic BLNPs (VMC and BDA) and SD-iCCAs, and no clinical follow up studies showing malignant transformation of unusual/dysplastic VMCs and BDAs to SD-iCCAs in the same tumors have been observed. So far, much of the evidence remains associative rather than causative, and precursor–carcinoma sequences of unusual/dysplastic BLNP have not been established. However, if precursors or early malignant lesions of SD-iCCA exist, they could be found in unusual/dysplastic BLNPs. So, it is imperative to narrow down or clarify which lesions of these BLNPs, particularly unusual/dysplastic VMCs or BDAs, are precursors.

#### 5.4.2. Relation of Dysplastic/Unusual BLNC with Low-Grade Malignant BLNP

Malignant transformation is suggested to develop in BAF, and AL-TCCs contained a distinct low-grade BAF-like component, suggesting that at least some AL-TCCs might have arisen in BAF [38]. These unusual/dysplastic VMCs and BDAs could be a risk factor or a precursor of AL-TCC and iCCA with DPMP [38]. In addition, as mentioned above, low-grade malignant BLNPs show a stronger association with VMC or DPM and biliary cysts and/or BDAs in the background livers [38], suggesting that unusual/dysplastic BLNPs and low-grade BLNPs may represent a continuum spectrum of biliary tumorigenesis. The components of conventional CCA are also found in low-malignant BLNPs, suggesting that low-grade malignant BLNPs could be a unique early malignant lesion followed by association with or development of conventional iCCA.

**Figure 6:**
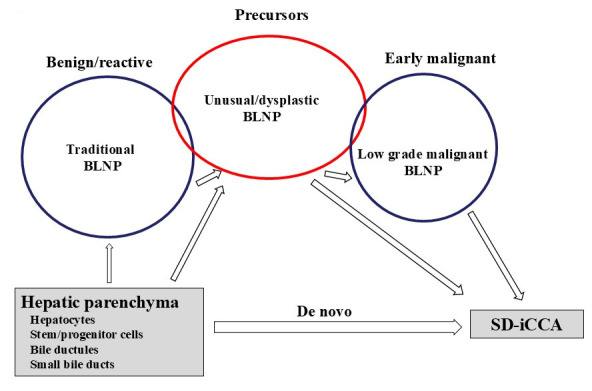
Relation among “traditional biliary lesions/neoplasm in hepatic parenchyma (traditional BLNP)”, “unusual/dysplastic BLNP”, and “low-grade malignant BLNP” is schematically depicted. Traditional BLNP lacks cytological atypia and does not seem to relate to small duct-type intrahepatic cholangiocarcinoma (SD-iCCA). Some unusual/dysplastic BLNP could be overlapped with traditional BLNP. Unusual/dysplastic BLNP may be followed by the development of SD-iCCA and may also be followed by the development of low-grade malignant BLNP. Some unusual/dysplastic BLNP could be overlapped with low-grade malignant BLNP. Low-grade malignant BLNP may be early malignant and may be followed by SD-iCCA. De novo carcinogenesis from hepatic parenchyma to SD-iCCA could be present.

The hypothetical relations of these three categories are schematically shown in Figure 6. This classification is comprehensive, ambitious, and promising, but still lacks a clear hierarchy and remains a future objective, at the moment. The implementational basis for a classification tool in practice still needs to be worked out.

## 6. Conclusions

In addition to (i) traditional benign lesions/neoplasms in the hepatic parenchyma (traditional BLNP) such as VMC and BDA in normal livers, recent studies have reported (ii) unusual/dysplastic VMC and BDA, and BAF with dysplasia (unusual/dysplastic BLNP), and (iii) SD-iCCA with DPMP and AI-TCC showing the reminiscent features of VMC, DPM, BAF, and invasive growth (low-grade malignant BLNP). Traditional BLNPs are thought to be a benign category. While candidate precursor lesions of SD-iCCA could be included in unusual/dysplastic BLNPs, which lesions of unusual/dysplastic BLNPs could be precursors of SD-iCCAs remain to be clarified. Low-grade malignant BLNPs could be associated with or followed by conventional SD-iCCAs. This novel approach to categorizing BLNPs into three groups guarantees further studies of precursors of and their progression to conventional SD-iCCAs.

## Figures and Tables

**Table 1:** Benign non-hepatocellular epithelial lesions and neoplasms in the hepatic parenchyma.

	Frequency	Relation to SD-iCCA
Benign biliary solid and solid/spongy-cystic lesions		
• von-Meyenburg complex	Common	Possible
• Bile duct adenoma	Occasional	Possible
• Biliary adenofibroma	Rare	37~50%
Bening biliary cystic lesions		
• Simple hepatic cyst	Common	None
• Polycystic liver disease	Occasional	None
• Multicystic biliary hamartoma	Rare	None
Benign ectopic or heterotopic lesions		
• Endometric cyst	Rare	None
• Ciliated hepatic foregut cyst	Rare	None

SD-iCCA, small duct-type intrahepatic cholangiocarcinoma.

**Table 2:** Characteristic features of representative biliary lesions/neoplasms in the hepatic parenchyma (VMC, bile duct adenoma, and biliary adenofibroma).

	VMC	Bile Duct Adenoma	Biliary Adenofibroma
Location	Any site, multiple (adjacent to portal tract)	Subcapsular, solitary	Subcapsular, solitary (occasionally any site)
Size	<0.5 cm	<1 cm	1.7–16 cm
Shape of cell	Flat—cuboidal	Cuboidal-low columnar	Cuboidal-low columnar
Nuclear/cellular atypia	Absent	Absent	Present (mild to marked)
Pattern of glands and their sizes	Variable sized glands with focally dilated lumina and microcystic changes	Uniform and small ductules with small or little lumen	Variable:tubular/mirocystic/cystic/papillary
Inspissated bile/proteinous fluid in lumen	Present, variably	Absent	Absent
Inflammation	Absent	Present, variable	Present, mild
Fibrous stroma	Present	Present	Present, moderate to marked
Invasive growth	Absent	Absent	Absent
Mucin	Absent	Acidic mucin of gastric type in the cytoplasm and luminal surface	Absent

VMC, von Meyenburg complexes.

**Table 3:** Representative hepatobiliary non-neoplastic lesions and neoplasms related to or mimicking DPM.

	Distribution of Lesions Related to or Mimicking DPM	Size of Lesions Related to or Mimicking DPM
Non-neoplastic disease		
• Caroli’s disease	Diffuse in the liver	Gross with or without microscopic
• Congenital hepatic fibrosis	Diffuse in the liver	Microscopic
• von Meyenburg complexes	Focal and scattered in the liver	Microscopic
Neoplastic disease		
• Biliary adenofibroma	A whole to considerable areas of tumor	Gross and microscopic
• iCCA with DPMP	A whole to considerable areas of tumor	Microscopical lesion
• AL-TCC	Variable areas of tumor	Gross and microscopic

DPM, ductal plate malformation; iCCA, intrahepatic cholangiocarcinoma; AL-TCC, adenofibroma-like tubulocystic cholangiocarcinoma.

**Table 4:** Biliary lesions/neoplasms in the liver different from VMC, bile duct adenoma, and biliary adenofibroma.

	Bile Ductular Reaction/Parenchymal Extinction	Peribiliary Glands	Well-Differentiated Cholangiocarcinoma,
Location and shape	• Any site in the liver, several to multiple Some are not circumscribed Some are circumscribed (extinction)	• Around intrahepatic large bile ducts • Several to multiple, acinar and lobular	• Any site in the liver • Single to multiple, nodular, circumscribed
Size	• Smaller size, usually microscopical	• Microscopical	• Any size
Shape of epithelial cell	• Cuboidal-low columnar	• Cuboidal-low columnar	• Flat—cuboidal- columnar
Nuclear/cellular atypia	• Absent	• Absent	• Minimum—mild
Gland pattern	• Uniform and small ductules	• Uniform and small Serous acini; main Mucinous acini, few	• Variable gland pattern in size and shape
Inspissated bile	• Absent—present (occasional and focal)	• Absent	• Absent
Inflammation	• Present, minimal—marked	• Absent	• Absent—present (variable)
Fibrous stroma	• Present, minimal	• Embedded in fibrous tissue	• Absent ~ present (variable)
Invasive growth	• Absent	• Absent	• Present, mild—marked
Mucin	• Absent	• Positive in mucinous acini • Negative in serous acini	• Absent to present (variable)
Ki-67 labeling index	<5%	<5%	>10%

VMC, von-Meyenburg complex.

**Table 5:** Differential diagnosis of VMC and bile duct adenoma and two mimickers ().

	VMC	Bile Duct Adenoma	Well-Differentiated Cholangiocarcinoma, Intrahepatic, Small Duct-Type	Ductular Reaction/Parenchymal Extinction
Size	<0.5 cm	<2 cm	Any size	Any size
Histological				
Shape of cell	Flat~cuboidal	Cuboidal	Flat~cuboidal, columnar	Flat~cuboidal
Nuclear atypia	Absent	Absent	None~mild	Absent
Gland pattern	Glands more variable in size with focally dilated lumina	Uniform small ductules with little or small lumen	Variable gland pattern in size and shape	Uniform small ductules with little or no lumen
Inspissated bile	Present, focal	Absent	absent	Absent~present, focal
Inflammation	Absent	Absent~present, mild	absent~present, mild	Present: minimal~marked
Hyalinized stroma	Present	Absent~present, mild	Absent~present, mild	Present, minimal
Invasion	Absent	Absent~present, minimal	Present, mild~marked	Absent
Immunohisto- chemical				
Cytokeratin (CK)	CK7, CK19	CK7, CK19	CK7, CK19 CK20 (20%)	CK7, CK19
CEA	Absent	Absent	Absent~present	Absent
MUC mucin	MUC1 (focal), MUC6 (focal)	MUC6 (focal), MUC5AC (focal)	MUC1, MUC6, MUC5AC (focal)	MUC6 (focal)
Ki-67 labeling index	<5%	<5%	>10%	<5%
p53	Absent	Absent	Absent~present	Absent
ARID1A (loss)	Absent	Absent	Absent~present	Absent
p16^INK4a^	Present	Present	Absent	Present
EZH2	Absent	Absent	Present: minimal~marked	Absent
IMP3	Absent	Absent	Present: minimal~marked	Absent

VMC, von-Meyenburg complex.

**Table 6:** Features characterizing usual and unusual bile duct adenoma.

Findings	Usual	Unusual Features
Location	• Subcapsular regions	• Deeper in the hepatic parenchyma • Around large caliber bile duct
Incorporation of normal portal tracts	• Present, particularly in the peripheral parts	• Absent
Multiplicity	• Solitary	• Several to multiple, usually associated with chronic liver diseases • BRAF-associated multiple BDA (adenomatosis)
Background liver and association of malignancy	• Normal liver	• Chronic advanced liver diseases • α1-antitrypsin deficiency • Small duct-type intrahepatic cholangiocarcinoma
Stroma	• Fibrotic and inflammatory stroma	• Compact growth of tubular components with few stroma
Sizes	• Usually less than 1 cm	• Larger than 2 cm
Microcystic changes	• Absent	• Present
DPM	• Absent	• Admixed
Mucin in supra-nuclear or luminal regions	• Variable, usually considerable	• Absent
Uniformity	• Uniformly shaped and sized tubular component	• Irregular
Nuclear changes	• Small and regular and benign	• Mild dysplastic

DPM, ductal plate malformation.

**Table 7:** Categorization of representative biliary lesions/neoplasms in hepatic parenchyma referring to VMC, bile duct adenoma, and biliary adenofibroma into three groups.

	VMC, VMC-Related, and DPM-Related Lesions/Neoplasms	BDA and BDA-Related Lesions/Neoplasms	BAF
Traditional BLNP	• VMC	• BDA	• BAF without cytological atypia, if exist but was not reported
Unusual/dysplastic BLNP	• VMC frequently found in background liver of SD-iCCA • Dysplastic VMC	• Unusual BDA (see Table 6) • BDA with the *BRAF V600E* mutation	BAF with mild to severe dysplasia
Low-grade malignant BLNP	• SD-iCCA with DPMP	• Not reported	• TCC with BAF-like features (AL-TCC) • BAF with foci of carcinoma showing DPM or BDA

VMC, von Meyenburg complex; BLNP, biliary lesions/neoplasms in hepatic parenchyma; BDA, bile duct adenoma; BAF, biliary adenofibroma; DPM, ductal plate malformation; DPMP, DPM pattern; SD-iCCA, small duct-type intrahepatic cholangiocarcinoma.

## Data Availability

In this review paper, no new deata were created.

## Abbreviations

The following abbreviations are used in this manuscript:

AAT	α1-antitryupsin
AL-TCC	tubulocystic carcinoma with biliary adenofibroma-like lesions
BAF	Biliary adenofibroma
BBLN	Benign biliary lesions/neoplasms
BDA	Bile duct adenoma
BilIN	Biliary intraepithelial neoplasm
CCA	Cholangiocarcinoma
CK	cytokeratin
CoCC	cholangiolocellular carcinoma
DCIS	ductal carcinoma in situ
d-PAS	PAS after diastase digestion
DPM	Ductal plate malformation
DPMP	Ductal plate malformation pattern
EMA	epithelial membrane antigen
HE	hematoxylin and eosin
iCCA	Intrahepatic cholangiocarcinoma
IPNB	intraductal papillary neoplasm of bile duct
LD-iCCA	Large duct-type intrahepatic cholangiocarcinoma
LOH	loss of heterozygosity
MCBH	Multicystic biliary hamartoma
N-DPMP	Neoplastic ductal plate malformation pattern
SD-iCCA	Small duct-type intrahepatic cholangiocarcinoma
TCC	tubule-cystic carcinoma
VMC	von-Meyenburg complex
